# Psoriatic Arthritis during Treatment with Bevacizumab for Anaplastic Oligodendroglioma

**DOI:** 10.1155/2012/208606

**Published:** 2012-11-29

**Authors:** D. Graceffa, E. Maiani, A. Pace, F. M. Solivetti, F. Elia, C. De Mutiis, C. Bonifati

**Affiliations:** ^1^Centre for the Study and Treatment of Psoriasis, Department of Clinical Dermatology, San Gallicano Dermatological Institute IRCCS, 00144 Rome, Italy; ^2^Department of Neuroscience, Regina Elena National Cancer Institute, 00144 Rome, Italy; ^3^Radiodiagnostic Facility, San Gallicano Dermatological Institute IRCCS, 00144 Rome, Italy

## Abstract

Bevacizumab is a recombinant humanised monoclonal antibody directed against the vascular endothelial growth factor (VEGF). The drug, alone or in combination with other anticancer agents, has been shown to be effective against several types of neoplasms. We report a case of a woman with a history of severe psoriasis who developed psoriatic arthritis during a course of bevacizumab, which was administered for a malignant glioma.

## 1. Introduction

The growth of new blood vessels from preexisting vessels (angiogenesis) plays a crucial role both in neoplastic and inflammatory processes [1]. Vascular endothelial growth factor (VEGF) is a key mediator of angiogenesis and its selective inhibition by bevacizumab (Avastin), a humanized monoclonal antibody, has been reported to be beneficial as therapy for several types of cancers, including malignant gliomas [2, 3]. Moreover bevacizumab has been shown to exerts anti-inflammatory properties at the level of the retina after intravitreal injection in diabetic patients affected with proliferative diabetic retinopathy [4]. Here we describe for the first time the case of a patient with a history of severe psoriasis who developed psoriatic arthritis (PsA), a typical inflammatory disease, during a course of bevacizumab therapy. 

## 2. Case Report

In March 2012, a 43-year-old woman was referred to our psoriasis outpatient clinic because of the tenderness of the temporomandibular joints and painful swelling of the left thumb, with onset two weeks earlier. In September 2011, the woman had begun treatment with bevacizumab (540 mg every 3 weeks) for an anaplastic oligodendroglioma of the left temporal lobe; when she presented to our clinic, she had already received the 6th infusion of bevacizumab. The tumour had been partially removed at the time of diagnosis in 2001, when she was 33 years old. Before starting bevacizumab therapy, the woman had undergone several types of therapy for oligodendroglioma, such as radiotherapy, temozolomide, and fotemustine, yet with only a partial reduction of the tumour. 

The woman had a history of psoriasis, which was first diagnosed when she was 15 years old. As treatment, she underwent different topical therapies (corticosteroids, vitamin D analogues) and systemic therapies (PUVA, cyclosporine). When she presented to our clinic, the psoriasis had completely cleared, without signs of nail involvement.

**Figure 1:**
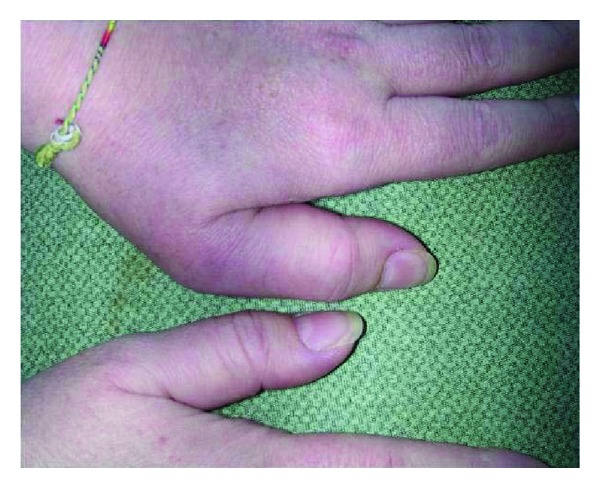
Dactyltis of the left thumb with the involvement of the metacarpophalangeal joint of the same finger.

At the physical examination, the woman presented with severe pain in the temporomandibular joints, which greatly limited her in opening her mouth. A dactylitis of the left thumb was also observed, together with the involvement of the metacarpophalangeal joint of the same finger (Figure 1).

**Figure 2:**
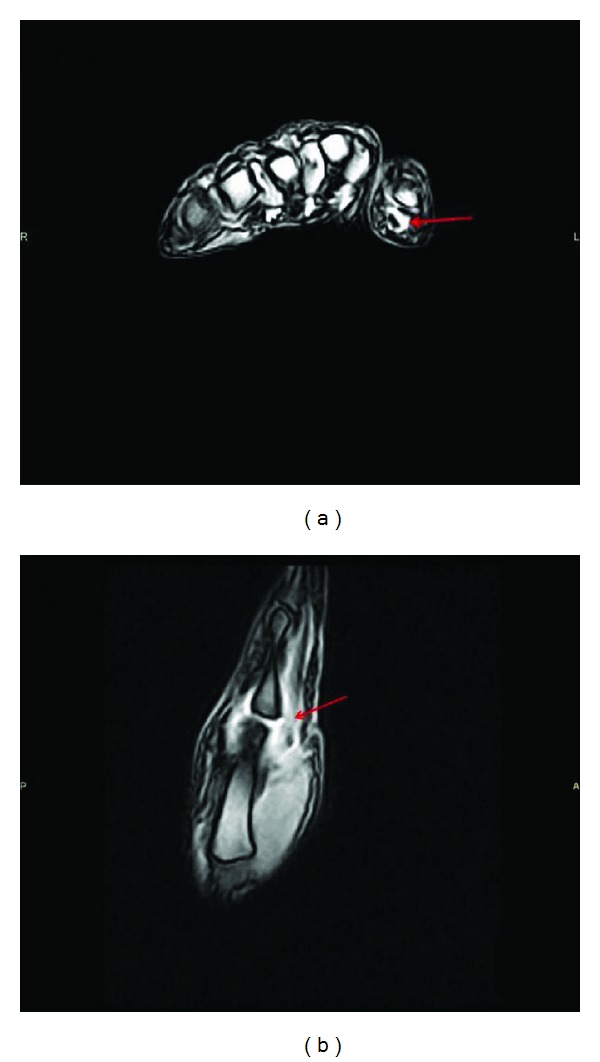
(a) T2 weighted MRI of the left hand shows a fluid distension of the sheath of the flexor tendons of the thumb. (b) T2 weighted MRI of the left hand shows fluid distension of the joint capsule of the 1st metacarpophalangeal.

At this level a plain radiograph was negative, whereas magnetic resonance showed the fluid distension of the joint capsule associated with the fluid distension of the sheath of the flexor tendons (Figure 2). Laboratory tests (blood count, ESR, CRP, C3, C4, ANA, rheumatoid factor, gammaglobulins, and urinalysis) were all negative or within the normal range; the only exception was positivity for anticitrullinated protein antibodies (37.8 U/mL; nv: 0–5 U/mL). Based on the clinical and instrumental findings, the woman was diagnosed with inflammatory arthritis, which was classified as “psoriatic” in accordance with CASPAR criteria [5].

Because bevacizumab was the only drug that the woman was taking when she developed PsA, treatment was discontinued, and a course of prednisone (25 mg/day) was started. After 15 days of corticosteroid therapy, the joint symptoms improved and there was a reduction in the dactylitis. Prednisone was tapered off and discontinued after 1 month of therapy, with a complete resolution of the joint pain and persistence of mild dactylitis.

## 3. Discussion

Of the adverse events associated with bevacizumab therapy, PsA has never been described. Whether or not the occurrence of PsA was only a coincidence or whether the therapy played a causative role cannot be established, and there are no data available to justify the occurrence of this phenomenon.

The onset of typical PsA (olygoarthritis and dactylitis) in an individual with a history of psoriasis during therapy with an antiangiogenic drug such as bevacizumab seems to be paradoxical. In fact, angiogenesis appears to be a first-order event in PsA as well as in psoriasis [6]. Moreover, a dramatic improvement in psoriasis in an individual treated with bevacizumab for colon cancer has been reported [7]. In conclusion, the case reported herein suggests that it is necessary to take into consideration the possibility of the onset of inflammatory arthritis when bevacizumab is administered in an oncologic patient who is also affected by psoriasis.
